# Neurological Tremor: Sensors, Signal Processing and Emerging Applications

**DOI:** 10.3390/s100201399

**Published:** 2010-02-24

**Authors:** Giuliana Grimaldi, Mario Manto

**Affiliations:** FNRS, Neurologie ULB-Erasme, 808 Route de Lennik, 1070 Bruxelles, Belgium; E-Mail: giulianagrim@yahoo.it

**Keywords:** tremor, sensors, signal analysis, brain-computer interface (BCI)

## Abstract

Neurological tremor is the most common movement disorder, affecting more than 4% of elderly people. Tremor is a non linear and non stationary phenomenon, which is increasingly recognized. The issue of selection of sensors is central in the characterization of tremor. This paper reviews the state-of-the-art instrumentation and methods of signal processing for tremor occurring in humans. We describe the advantages and disadvantages of the most commonly used sensors, as well as the emerging wearable sensors being developed to assess tremor instantaneously. We discuss the current limitations and the future applications such as the integration of tremor sensors in BCIs (brain-computer interfaces) and the need for sensor fusion approaches for wearable solutions.

## 1. Introduction

**Figure 1.**
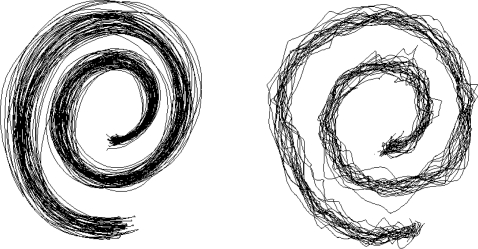
Superimposition of spirals drawn on a digitized tablet. Left panel: control subject, right panel: patient with tremor - the spirals are irregular with swerves.

Tremor is the most common movement disorder. Its incidence and prevalence increase with ageing, affecting more than 4% of the patients older than 65 years [1]. More than two-thirds of the population with upper limb tremor face serious difficulties in daily life. Although tremor is not life-threatening, it causes functional disability and social inconvenience, contaminating daily life activities, such as writing, pouring, eating, and so on [2]. Figure 1 illustrates an example of the effect of tremor in the spiral copying test (Archimedes’ spiral).

Tremor can be defined as a rhythmic shaking of a body part [3]. It occurs in healthy individuals as the so-called physiological tremor, which is composed of two distinct oscillations (mechanical reflex and central neurogenic) superimposed on a background of irregular fluctuations in muscle forces and displacements [4,5]. The mechanical reflex component is governed by the inertial and elastic properties of the body, whereas the central neurogenic component is associated with the modulation of motor unit activity under the control of active generators in the brain. Participating motor units usually discharge at about 8–13 Hz [6]. The central component is inertia-insensitive. Physiological tremor may be enhanced by anxiety, stress, fatigue and medications.

**Figure 2.**
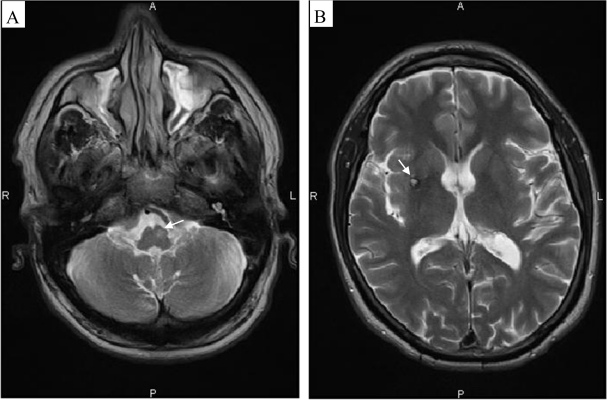
Brain MRI of a patient exhibiting a disabling neurological tremor in the upper limbs. Note the left inferior olivary hypertrophy (Guillain-Mollaret triangle; arrow in left panel, (A) and the lesion in putamen (arrow in right panel, (B). Axial T2-weighted images. R: right, L: left.

In neurological patients, tremor is clinically described as rest, postural and/or kinetic tremor [7] according to its mode of clinical presentation. Rest tremor appears during resting. Postural tremor is triggered by maintenance of a posture or a position against gravity. Kinetic tremor is evoked by a voluntary movement and is maximal near the target. In a clinical setting, tremor is characterized by its dominant frequency and its power spectral density. Rest tremor frequency is typically in the 3–6 Hz frequency range [7,8] and may increase with mental stress (*i.e.*, counting backwards) or contralateral voluntary motion [8]. The most common cause of rest tremor is idiopathic Parkinson’s Disease. The frequency of postural tremor is usually between 4 and 12 Hz. Many disorders are associated with postural tremor in upper limbs. Essential tremor is the commonest cause [9]. Kinetic tremor has a frequency between 2 and 7 Hz in the large majority of cases [7]. Tremor occurring in cerebellar diseases is the typical example of a kinetic tremor. Cerebellar disorders are either sporadic or genetic [10]. The cerebellum is considered to be a major site for tremorgenesis. Detailed lesion-mapping studies have now delineated the regions of the cerebellum that cause deficits in limbs or trunk [11]. Concerning imaging, brain MRI has become a routine procedure in the evaluation and follow-up of neurological patients [12]. Tremor may be associated with a single or multiple generators in the brain. Figure 2 illustrates the role of brain imaging in the identification of anatomical lesions participating in the generators of tremor.

Although tremor can be estimated clinically, the non stationary feature and the difficulties related to a pure clinical evaluation (with inherent subjectivity) make the use of sensitive, reliable and stable sensors mandatory. Repercussions on daily living activities (ADL) can be evaluated using specific questionnaires such as the ADL-T24, which has a good inter-session reproducibility (Table 1).

Clinical scales include the Tolosa-Fahn-Marin scale, as well as other specifically-designed and detailed composite scales combining clinical and functional evaluation such as the composite CNF-TES (clinical neurophysiological functional tremor evaluation scale; Table 2) which compares clinical, neurophysiological and functional results. Differential diagnoses of tremor includes dystonia (prolonged muscle contractions that lead to abnormal postures), dyskinesias (acute or chronic involuntary movements similar to tics, chorea, spasm, or myoclonus), chorea (irregular movements with a dance-like aspect), athetosis (continuous slow hyperkinesia of distal segments), tics (repetitive hyperkinetic movements usually with a head topography) and myoclonus (sudden and short movements). In essence, tremor is stereotyped and repetitive.

Several sensors for the monitoring and analysis of tremor have been developed these last decades (Table 3). The advantages and disadvantages of techniques for tremor evaluation are summarized (see also references [8], 15–17).

They provide information about which are complementary to the clinical evaluation and often guide therapies, hence their critical role in this field. The combination of electromyography (EMG) with kinematic sensors is widely used. Each technique will be described below.

### General recommendations

As a prerequisite, any biomedical instrumentation, independently from its technical characteristics, requires the appropriate placement of the sensor on the body segment and a quiet recording environment free of electrical interferences. Regular calibration of the instrumentation should not be underestimated. Standardized recording procedures are essential for intra- and inter-patients comparisons [8]. Both the clinical examination and quantification of tremor should be performed by the same examiner if possible, in order to reduce the anxiety effect, which may cause noticeable differences in results [18]. Tremor recordings should be performed immediately after clinical examination. Functional tests provide useful additional information for a better understanding of patients’ disabilities. Indeed, functional evaluation is now part of the evaluation of neurological tremor. The need for blind or even double blind studies when possible, as well as future studies with control conditions should be underlined.

## 2. Sensors and Instrumentation for the Characterization of Tremor

The issue of the localization of tremor generators remains central in the field of tremor research [19]. The selection of the sensor helps in improving (1) our understanding of the respective contributions of supra-spinal and spinal structures, and (2) how the state of the neuromuscular periphery influences tremor. Using sensors that do not significantly impede wrist movements (so-called ‘soft’ tremor sensors) and more rigid sensors (approximating the condition to isometric), it has been shown that the fixation of a wrist joint decreases the amplitude of tremor bilaterally with a relatively low impact on tremor frequency [19], suggesting an important role for the neuromuscular and spinal cord mechanisms in determining tremor amplitude. This is an example of how mechanical properties of a sensor influence results and interpretation of the mechanisms of tremor.

### 2.1. Electromyography (EMG)

**Figure 3.**
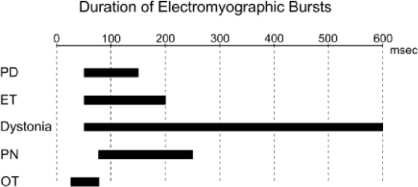
Duration of EMG bursts in forearm muscles in various neurological disorders associated with tremor. Abbreviations: PD: Parkinson’s disease, ET: essential tremor, PN: peripheral neuropathy, OT: orthostatic tremor. Adapted from Grimaldi and Manto, 2008.

**Figure 4.**
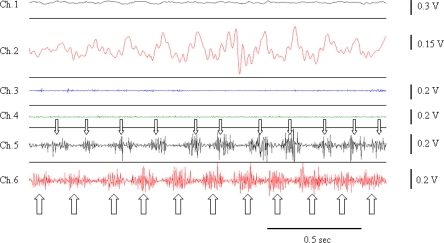
Recordings of action tremor in a neurological patient. Note the oscillations in channel 2 (accelerometry, right side; accelerometer fixed on the extremity of the index finger) and the alternating pattern of EMG bursts in channels 5 (Flexor carpi radialis, right side – small arrows) and 6 (Extensor carpi radialis, right side – large arrows). Channel 1, channel 3 and channel 4 correspond to accelerometry and EMG recordings on the left side.

Surface EMG (SEMG), needle EMG and recordings with fine wires record electrical potentials generated by the muscle fibers [20]. For SEMG, sensors are fixed on the skin at the level of muscle(s) of interest, using either self-adhesive disposable electrodes or differential electrodes with built-in pre-amplifiers. Several electrodes commercially available do not require conductive gel or skin preparation. Surface electrodes are arranged approximately 2 cm apart over the muscle. The use of anatomical landmarks is recommended to avoid variations due to electrode misplacement. Regarding intra-muscular studies with conventional needles or fine wire electrodes, electrodes are implanted in a given muscle to extract the properties of motor unit firings (firing times, firing rates, recruitment threshold, cross-correlation of firing rates of concomitant motor units). Analysis of the duration of bursts of EMG activities can be useful to distinguish the various disorders (Figure 3). Patterns of muscle contractions (co-contraction/alternating) are identified with EMG recordings (Figure 4).

Patterns of motoneuronal discharges might constitute “motoneuronal signatures” which takes into account the size of the recruited motor units, the timing of their respective firings, their firing rates and their modulation [21]. Interestingly, tremor recordings during a tapping exercise with the controlateral arm provide a quantified electrophysiological entrainment test which is a sensitive and specific procedure to distinguish psychogenic tremor from dystonic and other organic tremors [22].

**Figure 5.**
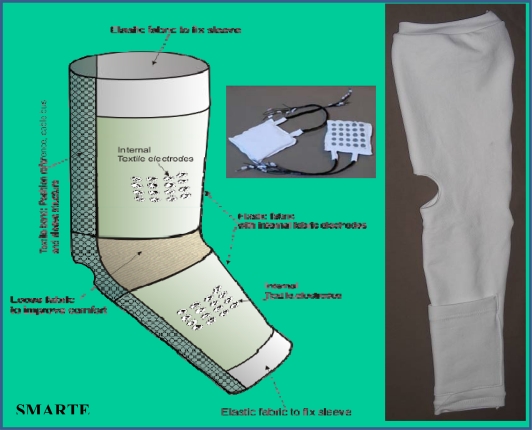
Illustration of a textile with a matrix of surface EMG electrodes used to record upper limb tremor. The textile is designed to fit with comfort issues. Courtesy of Smartex, Italy.

Long-term EMG has proven to be a valid and reliable method for the quantification of pathological tremors. Recent works indicate that these recordings allow a rater-independent classification of parkinsonian *versus* essential tremor [23]. Arrays of SEMG are being adopted by many laboratories. The arrays can be integrated in textiles for wearable solutions (Figure 5), with the aim of combining spatio-temporal accuracy with aesthetic requirements and comfort for daily use.

### 2.2. Accelerometers

Accelerometer measures acceleration along the sensitive axis of the sensor based on Newton’s second law (Force = Mass x Acceleration). The loading force drives a second-order damped harmonic oscillator in a mass-spring-damper system [15]. There are three main categories of accelerometers: piezoelectric, piezoresistive and capacitive types. Impedance, signal level, effects of gravitational component and cost are some important parameters related to this technique [15]. Table 4 compares the three types of accelerometers. The natural high-pass filtering of piezoresistive accelerometers reduces significantly slow drifts [24]. The need for a second integration to obtain displacement values and the need for regular calibration are considered as negative aspects. Another disadvantage is the fact that accelerometers measure linear acceleration, while articular motions are mainly based on rotations of joints. Data from accelerometers are composed of a combination of linear acceleration, gravity, and additive noise [16]. There is still no validated analytic model to distinguish between data due to acceleration and gravity. Low-pass filtering is commonly used [25].

**Figure 6.**
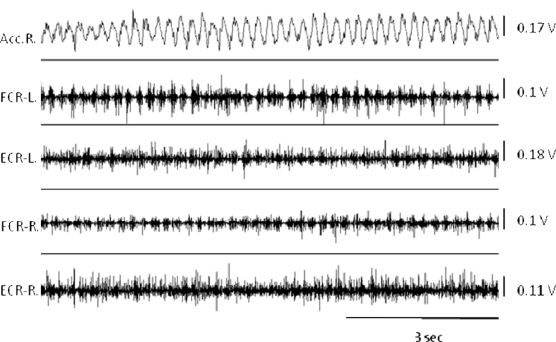
Example of tremor in a patient with multiple brain lesions. One single-axis accelerometer is affixed on the extremity of right index (Acc. R.) while the patient is performing the finger-to-finger test (the index fingers are maintained horizontally at a distance of about 1 cm in front of the patient). Surface EMG recordings of the flexor carpi radialis (FCR) and extensor carpi radialis (ECR) muscles are shown for both sides (L: left, R: right). Note the oscillations in the accelerometry and the regular bursts of EMG activities. Peak tremor frequency: 4.7 Hz.

Accelerometry is simple, relatively reliable and remains a convenient technique to measure frequency and amplitude of oscillations of body segments [7]. Sensors are fixed on the skin at given anatomical landmarks. An example of upper limb tremor is shown in Figure 6, recorded with a combination of accelerometry and surface EMG recordings.

Various levels of support of the limbs have been proposed [26]. The feasibility of using data recorded with accelerometers to estimate the severity of symptoms and motor complications in patients with Parkinson’s disease has been shown recently [27]. Accelerometers are also used for intra-operative assessment of the best position to implant electrodes for deep brain stimulation (DBS) and neurophysiological monitoring of stereotactic intervention of movement disorders [28]. Accelerometers allow the estimation of orientation of body segments, acceleration of trunk, acceleration/velocity/translations of limbs [8]. These are clinically useful parameters for the management of neurological patients. There are also applications outside human disorders, for instance in animal models of tremor [29]. The model of harmaline-induced tremor in rodents is an example.

### 2.3. Gyroscopes

A gyroscope can be defined as an angular velocity sensor. The angular orientation can be obtained from integration of the velocity signal [15]. Gyroscopes are based on the measurement of the Coriolis force of vibrating devices. Coriolis force is an apparent force arising in a rotating reference frame and proportional to the angular rate of rotation. There are three basic types of gyroscopes: spinning rotor gyroscope, ring laser gyroscope and vibrating mass gyroscope. The last one presents many advantages for portable applications due to its size, weight, lower power consumption and cost. An internal mass in constantly vibrating inside the sensor. Gyroscopes are being used to measure velocity and stride length, joint angle of lower limbs, angular velocity of trunk rotation, and angular displacement of trunk motions [8]. They are often selected for implementation in wearable exoskeletons. Gyroscopes are considered as presenting long term stability, eliminating the need for periodic recalibration. However, a disadvantage is the presence of a low frequency bias, mainly due to temperature effects. Gyroscope stability and behavior over variations in temperature is essential for the long-term performances. Low-cost “strap down” gyroscopes are now internally compensated in temperature and therefore the bias vector slowly oscillates around a constant average value [30].

### 2.4. Flexible Angular Sensors and Goniometers

Strain gauges are usually inserted in goniometers, in order to measure a joint angle dynamically. The electrical output is proportional to the angle. Flexible angular sensors are commonly used in rehabilitation and sports, but have not been disseminated at a large scale for studies of tremor [8,31,32]. Some goniometers include flexible springs to compensate for joint migration related to movement. Double differentiation of angular position is performed to compute joint torque T associated with oscillations of limbs, according to the following equation:

T=Jα

Where J is the limb moment of inertia and α is the angular acceleration.

### 2.5. Videos

Videotaping of patients and computerized video motion detecting systems are qualitative and quantitative methods useful of analysis of motion disorders [33]. Measuring devices based on video imaging are effective in quantifying motor impairments in clinical settings [34]. For instance, videos can be used to evaluate patients presenting Parkinson’s disease. However, the videotaped motor examination may present some limitations when patients have mild symptoms [35]. Experimental procedures for assessment of tremor as well as validation of new methods benefit from videotaping [36–37]. Videotaping is also a valuable teaching tool to improve the uniform application of tremor rating scales by raters having different levels of experience in movement disorders [38]. The use of at least two cameras is recommended to compute three-dimensional estimations. A calibration method, as well as a quiet room for recording are mandatory. It should be pointed out that analysis of videos may be time-consuming. This may become an obstacle in the evaluation of neurological disorders.

### 2.6. Optoelectronic Devices

Optoelectronic devices are popular in the field of motion analysis. Most optoelectronic devices use reflective (passive) or active markers (such as infra-red light emitting diodes LEDs) fixed on anatomical landmarks of the body, such as bone landmarks [39,40]. Although they provide accurate measurements and allow for multi-site recordings, the procedure is often time-consuming. Most of them require a calibration procedure. The estimation of three-dimensional positioning from digitized data is based on the geometrical properties of a central projection [41]. Recognition of passive markers can be performed either via recognition software or with a dedicated hardware circuit [42–44]. Active markers are detected thanks to detection of sequential pulses. The absence of wires, batteries and pulsing circuitry are advantages of passive markers, but accuracy of active markers is higher. An important drawback is due to marker visibility constraints. Also, instrumental errors can arise [42]. Calibration inaccuracies, image distortion, electrical noise, errors in positioning of markers and marker flickering (imprecision related to image conversion), as well as experimental mistakes related to the selection of the environment may occur [42,45]. Compensation methods that have been developed, such as least-squares techniques applied to coordinates of markers allow improvement of the bone pose estimate [42]. Skin deformation and displacement causes marker movement with respect to the underlying bone [46]. This soft tissue artefact has a frequency content similar to the actual bone movement, is task dependent and not reproducible among subjects [46]. For lower limbs, motion about the flexion/extension axis of hips, knees and ankles can be determined reliably, but motion about other axes at those joints should be interpreted cautiously, as the soft tissue artefact produces spurious effects with amplitudes comparable to the amount of motion actually occurring in those joints [46].

### 2.7. Force Sensors

Detailed instantaneous monitoring of force during functional tasks is promising both for the diagnosis and management of tremor. In the last decade, robotic devices have become more popular. They combine the haptic technology with conventional neurophysiological assessment including EMG (myohaptic devices). The haptic tools are also proposed as future therapeutical methods for rehabilitation [47]. These instruments record angular motion, torques and corresponding EMG activities (in case of combination with EMG sensors), and can be used to obtain real-time spectral analysis, to apply loads and mechanical oscillations as well as characterize the effects of damping on voluntary motion in neurological patients [48]. Comparison with conventional accelerometers has demonstrated a high accuracy in terms of reproducibility of frequency and amplitudes of imposed oscillations [49]. However, several haptic devices are non portable and expensive, two factors which limit their current dissemination.

Most clinical and instrumental methods evaluate tremor amplitude and frequency, without considering joint movement in the affected limb. Estimation of joint movement facilitates the identification of the muscle groups which directly contribute to tremor. Rozman et al. have developed a method based on force transducers to evaluate rest tremor of the wrist and metacarpophalangeal joints in two degrees of freedom for each joint [50]. This technique of direct measurements of joint movement in the hand could be particularly useful when therapies acting on selective muscles are considered, especially local botulinum toxin injections.

### 2.8. Evaluation of Handwriting and Drawing

Quantification of drawings of Archimedes’ spiral is either based on the off-line analysis of the digitized picture obtained from a commercial scanner [51], or from data collected from one or two accelerometers fixed on the pen. Spirals can decomposed and reconstituted, for subsequent analysis. The technique is relatively cheap and easy to assess two-dimensional tremor.

### 2.9. Wearable Orthosis

The field of wearable sensors in bioengineering applications and medicine is growing rapidly [52]. The impact of wearable sensors is anticipated to be high in tremor research, given the need to obtain accurate estimations of tremor parameters during daily life activities. Wearable sensing can be used to monitor motor difficulties, to estimate fluctuations of deficits with time and response to therapies. Parkinson’s disease is a typical disorder under investigation [53]. Fine adjustment of doses and comparison of modes of administration of drugs are examples of applications.

Quantification of tremor can be achieved using a wearable orthosis affixed on one upper limb [2]. An exoskeleton acting in parallel to the limb makes real-time estimation of upper limb tremor. In addition to measuring oscillations, this technique allows to investigate the effects of adaptative viscous control. Gyroscopes or accelerometers can be integrated in the orthosis for tremor monitoring.

The main disadvantages of the wearable orthosis are the issues of aesthetics, cosmetics and the difficulty to make them affordable for each individual. They require multiple shapes and sizes. Future studies are required for the integration of sensors and actuators in comfortable textiles designed in order to be accepted by users. Tremor suppression using smart textiles takes the advantage of material flexibility, not hindering the bearer’s motor functions [54]. Active and semi-active control devices have been proposed to suppress oscillations [54]. Wearable hybrid sensors are being developed, in particular sensors combining SEMG and gyroscopes. Studies on their reliability and stability are required.

### 2.10. Other Sensors

Sensors based upon magnetic fields (search-coil technique) are occasionally used for the characterization of tremor. Their main clinical application remains the assessment or upper limb kinematics [55].

### 2.11. Choice of the Sensor and Future Developments in the Assessment of Tremor

The choice of the sensors should be based upon their characteristics in terms of sensitivity to gravity, signal-to-noise ratio, bandwidth, size and weight (see Table 4) [8,30]. In addition, reliability and stability of the sensors used should be considered. For historical and practical reasons (size, price, ease of use), conventional accelerometers tend to be the most commonly used sensors [15]. They are disseminated in numerous laboratories worldwide. New advances in microelectromechanical sensors (MEMS are based on the integration of mechanical elements, sensors, actuators, and electronics on a common silicon substrate using microfabrication technology) are changing the field of sensors for tremor evaluation. The most used types of MEMS sensors are solid state accelerometers, gyroscopes, and also magnetometers. Since MEMS devices are being manufactured with batch fabrication techniques (similar to the techniques used for integrated circuits), high levels of functionality and reliability can be reached at a relatively low cost. However, because most of MEMS devices contain movable parts, material fatigue and aging under long-term repeated cycling load may cause potential device failure, which in turn decreases the device reliability [56]. Simulation results show that the fatigue lifetime of MEMS accelerometers made by poly-silicon material is relatively good. For MEMS gyroscopes, fabrication imperfections result in cross stiffness and cross damping effects that may interfere with the measurement of angular velocity [57]. Minimization of the cross coupling between two axes are challenging issues in vibrating gyroscopes. Several control algorithms have been proposed, including a sliding mode control with a force balancing control strategy and an adaptative controller for tuning the natural frequency of the drive axis of a vibratory gyroscope [58–60]. The proportional-integral sliding mode adaptative control proposed by Fei and Batur takes into account both matched and unmatched uncertainties and shows consistent estimation of gyroscope parameters including angular velocity and large robustness to parameter variations and external disturbances [57]. Overall, inertial sensing appears promising for tremor. Wireless inertial sensor with the combination of different types of sensors (for instance three linear accelerometers, three gyroscopes and three magnetometers [61]) is now being applied.

**Figure 7.**
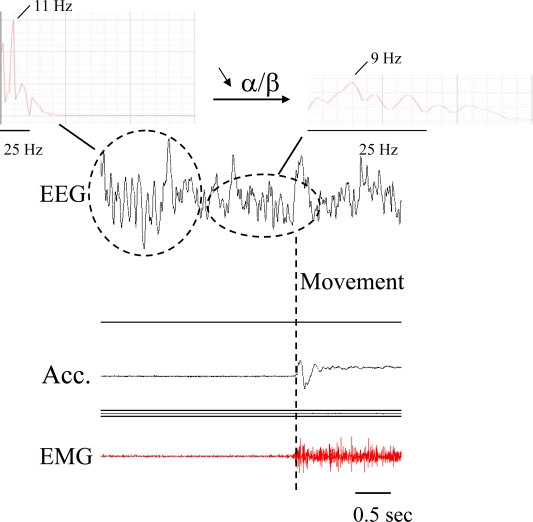
Example of change in alpha rhythm recorded with needle EEG on the scalp before a distal movement in contralateral upper limb. Preparation of movement is associated with a desynchronization of the EEG rhythm, with a decrease in the alpha/beta ratio. Upper panels correspond to power spectrum of EEG segments. Acc: accelerometer fixed on the hand; EMG: surface EMG in forearm muscle. Dotted vertical line: movement onset.

A different approach to the direct measurement of trembling legs or arms is to use the EEG signal in the framework of a Brain Computer Interface (BCI) [62]. BCIs aim at providing a communication channel between brain and the external environment. BCIs can be either invasive or non-invasive. Invasive recordings relate to the recordings of brain electrical activity at the surface of the cortex (electrocorticography) or directly in the cortex (recordings of action potentials or local field potentials), whereas non-invasive recordings are obtained from the scalp by analysing electrical activity (EEG) or magnetic activity (magnetoencephalography) [63]. Most of the current BCIs are EEG-based, since EEG recordings have a high temporal resolution and can be portable, avoiding the need for implants. Control signals include movement-related cortical potentials (MRCP), slow cortical potentials, sensorimotor rhythms (SMR) and steady-state visual evoked potentials (SSVEP) [63]. For MRCP, a surface negativity occurs about two seconds before the movement onset (BP: Bereitschaftspotential). The amplitude of MRCP is influenced by the characteristics of the movement performed. Changes in SMR at the alpha band/beta band correspond to event-related desynchronization (ERD) and event-related synchronization (ERS), respectively for a power decrease and a power increase. An example is shown in Figure 7. Research is going on to extract the best parameter(s) from the BCI to detect tremor onset and differentiate tremor from voluntary movements initiated by patients. Combinations of ERD/ERS, with parameters extracted from instantaneous assessment of muscle discharges and changes in corticomotor coherence are being considered.

The ERD begins up to two seconds before movement onset. MRCP and ERD/ERS take their origin from different brain sources [64]. The early BP might reflect slowly increasing cortical excitability and subconscious readiness for the forthcoming movement [64]. Pattern recognition techniques have been proposed for the classification of single-trial MRCPs, based upon optimization of wavelets and support vector machine (SVM) [63]. SVM has shown good generalization property in various applications, including BCI [63].

Recent inter-disciplinary studies aim to achieve the early detection and monitoring of tremor through a multimodal brain computer interface (BCI) combining neurophysiological (EEG, EMG) and biomechanical data (MEMS inertial sensors) in a sensor fusion approach [65]. Estimation of upper-limb orientation using miniature accelerometers and gyroscopes is accurate and renders the measurement system less obtrusive [66]. The use of low-mass sensors is important to decrease the low-pass filtering effect due to mass addition. Both the sensor fusion approach and hybrid sensors offer the possibility to extract and combine the most reliable data from the respective instruments (EEG and EMG, EMG and accelerometers, EEG and gyroscopes,...). Sensors are intercommunicative, usually with the wireless technology. It is anticipated that BCIs will be used in the near future to distinguish voluntary motion from involuntary oscillations of the body in order to trigger an external device reducing tremor (namely exoskeleton applying viscosity, myohaptic device applying damping, neuromuscular functional electrical stimulation FES).

## 3. Methods of Signal Processing for Tremor

Tremorous activity is composed of deterministic (non random) and stochastic components. Signal processing is required to interpret time-series data of nonlinear systems and instances in which the frequency content of a signal provides more information than the original waveform [8].

### 3.1. Editing

Editing remains a critical step in digital processing of tremor series [8,67,68]. Data editing can be compared to a pre-analysis procedure allowing the detection and removal of low quality signals (DC offset removal,…). For tremor recordings, noise may result from a problem which occurred during acquisition (instrumentation error, recording in a noisy environment, low frequency noise due to movement artefacts related to tremor,…). Many experts edit through visual inspection of recordings presented graphically. Automatic detection of tremor is under development. A non-linear analysis technique based on a running second order moment function (SOMF) has been suggested [68]. Alternatives include the use of extended Kalman filter (EKF) to estimate the instantaneous tremor frequency, as applied in spike trains detected from microelectrode recordings [69]. The use of normalized variance of intensity of the signal adapted from studies of pulses in radio waves could be an alternative to identify the oscillations of tremor.

### 3.2. Noise Minimization and Wavelets

Noise minimization is generally required for tremor time series [8]. Frequency-selective filters or adaptative filters are used. Wavelet (time-scale distribution) denoising may be applied also to band-pass filter a given signal. Wavelet transforms co-localize in both frequency domain and time domain and are effective for non stationary signals such as neurological tremor [70]. The basic idea behind wavelets is to express a signal as a linear combination of given sets of functions (wavelet transform). These are obtained by shifting and dilating a function called mother wavelet. Because the signal in tremor has often many transient components which are interesting to isolate and analyze and due to noisy components, wavelet-based denoising can efficiently isolate activities of interest such as EMG discharges. Wavelet denoising allows a separation of the signal from noise [8].

### 3.3. Spectral Estimations

Most spectral estimation algorithms are devised for complete data sets. However, missing samples may occur. Non parametric adaptative filtering-based tools are now available to deal with missing-data samples. The “Gibbs phenomenon” relates to errors at points of discontinuities (a value of about 9% occurs in any region of discontinuity) [8]. A discrete Fourier transform (FT) is often used for signals composed of data sampled at given spaced intervals and a continuous signal may be reconstructed without information loss if the sampling frequency is greater than twice the highest frequency component in the signal (Nyquist critical frequency), to avoid aliasing. Direct application of the FT is typically modified by windowing. Spectra are averaged in order to reduce the variance of spectral estimates [8].

The spectral estimation methods allowing the calculation of the power spectra are the autocorrelation function, the Fast Fourier Transform (FFT) and the autoregression. Units for power spectra are in power per frequency band (e.g., (m/sec^2^)^2^/Hz). Results of spectral densities may be gathered in a time-frequency representation, which provides interesting and meaningful information for daily clinical practice [8]. Fourier transforms break down time-domain signals into constituent sinusoids of different frequencies. For periodic functions, the original waveform may be reconstructed from the sinusoidal components by application of the FT. The FFT is the most popular method to perform transformation in the frequency domain, being computationally simple and fast [24].

### 3.4. Parameters Commonly Extracted for Spectral Estimations

For human tremor applications, the following parameters are extracted [71–73]:

-spectrum shape with identification of single or multiple peaks
-peak frequency
-median frequency
-power spectral density (PSD), in particular in the band 1–20 Hz (PSD_1-20Hz_) and 1–33 Hz (PSD_1-33Hz_), with identification of the peak intensity (PI)
-power in specific frequency bands (α band: 8–13 Hz, β1 band: 13–20 Hz, β2 band 20–26 Hz; β3 band: 26–33 Hz) and power ratios for each frequency band as compared to total PSD
-crest factor: ratio of the PI divided by PSD_1-20Hz_ or PSD_1-33Hz_
-center frequency (F50): frequency of the power spectrum dividing the area under the spectrum in two equal parts
-harmonic index (HI): index comparing the tremor frequency pattern with the pattern of single harmonic oscillation (HI = 1 in case of single harmonic)
-frequency dispersion: corresponds to center frequency ± SD. Frequency dispersion is low for regular tremors.

The PSD is usually averaged over several epochs or smoothed over frequencies for reducing the variance of estimation, as explained above. The spectral content of the signal is presented in either linear or logarithmic scale. The FFT takes advantage of the symmetrical properties of sinusoidal periodic waveforms. Using FFT analysis the signal is linearly decomposed as combination of sines and cosines, but most tremors are non periodic. The compromise between time and frequency resolution of these methods may not underline the presence of local oscillations in the signal, which might bring key information for the understanding of tremor. Two additional techniques have been shown to provide useful information for human tremor. First, analysis of short data segments can be performed with an autoregressive method of spectral analysis. This is interesting for the analysis of individual movements. Second, the empirical mode decomposition (EMD) can be used to isolate tremor movements from non oscillatory movements [74]. EMD decomposes time-series into intrinsic mode functions (IMFs), whose energy is quantified.

Cross-spectral studies are widely used to study the relation between signals which are recorded simultaneously from distinct sources [8]. In particular, the relations between EMG, EEG and MEG have been investigated in details. The estimation of the *power spectrum* of a zero mean process *X_(t)_* is defined as the Fourier Transform (FT) of the auto-covariance function and is performed by a direct spectral estimation based on the FT of the measured data [8]. The *cross-spectrum* *CS(ω)* of two zero mean process *X_(t)_* and *Y_(t)_*, similarly to the univariate case, is defined as the FT of the cross-correlation function:

$${\mathrm{CCF}}_{\left(t\prime \right)}=\left(X_{\left(t\right)}\;Y_{\left(t-t\prime \right)}\right)$$

The modulus of the cross-spectrum *CS(ω)* normalized by the respective auto-spectra *Sx(ω)* and *Sy(ω)* gives the *coherence-spectrum*:

Coh(ω)=|CS(ω)|/√Sx(ω) Sy(ω)

The *coherence* can be seen as a measure of linear predictability. Coherence value is 1 whenever X*_(t)_* is obtained from Y*_(t)_* by a linear operator. Coherence value is equal to zero whether there is no relation or the relation between the processes is a quadratic one; interpretation of coherence does not rely on the linearity of the processes X*_(t)_* and Y_*(t).*_ Simultaneously recorded signals (X*_(t)_* and Y*_(t)_*) may be uncorrelated or present a coherence unequal 1 due to a non linear relationship, additional influences on X*_(t)_* apart from Y*_(t)_*, estimation bias or noise.

## 4. The Need for Sensors Providing Tremor Parameters Correlated with Clinical Scores and Functional Evaluation

**Figure 8.**
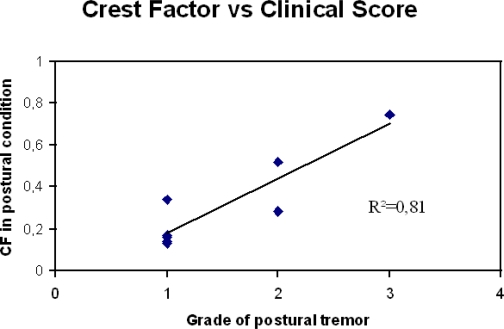
Correlation between the clinical grade of postural tremor and crest factor obtained with accelerometry during a postural task in a group of eight patients presenting a neurological tremor. Accelerometer fixed on the extremity of the index finger. Sampling rate: 512 Hz, duration of acquisition: 30 seconds.

In order to render sensors popular in the clinical community, several criteria need to be met. First, the sensors must be designed to fit with the clinical environment and the needs of users. This takes into account aesthetics and cosmetic criteria. Second, data should be correlated as much as possible with current clinical standards in terms of neurological evaluation. Figure 8 illustrates an example of a good linear correlation for upper limb tremor between the crest factor and the clinical score in a widely used clinical manoeuver.

**Figure 9.**
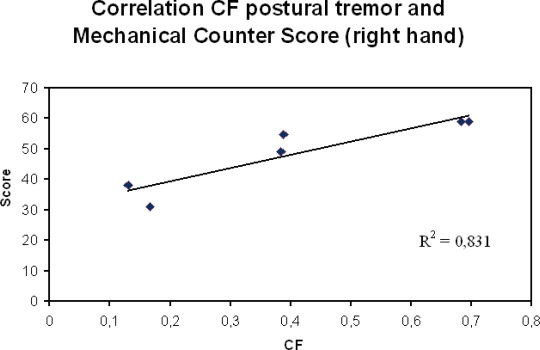
Correlation between the crest factor (CF) during maintenance of a postural task and the score obtained using mechanical counters (repeated tapping between 2 counters separated by 30 cm during 30 seconds) in a group of six patients presenting a neurological tremor. Accelerometer fixed on the extremity of the index finger. Sampling rate: 512 Hz, duration of acquisition: 30 seconds.

Third, data obtained should be correlated with functional scores. Figure 9 shows an example. The assessment of neurological patients is greatly improved when clinical scores, functional evaluation and parameters extracted using sensitive sensors are combined, especially for the monitoring of patients. The need for reliable sensors will increase in the future, because new therapeutic strategies for disorders previously considered as not treatable are emerging [75,76].

It should be underlined that the fact that sensor data should be correlated with clinical scores does not necessarily mean that a linear dependence is necessary. However, the pure clinical evaluation of tremor is often considered as a good clinical correlate of tremor severity, despite the variability of tremor in a given patient and amongst different patients [8,67]. Frequency dispersion is an example of a parameter which is not correlated with clinical staging of tremor. Patients may exhibit a strong variability in this parameter, despite a relatively stable crest factor and a high inter-observer reliability for the clinical scores, especially in postural tasks.

## 5. Conclusions

Neurological tremor is a disabling and common symptom related to various disorders, such as Parkinson’s disease, essential tremor or cerebellar ataxias. Its assessment, mainly based on popular sensors such as accelerometers and EMG, is critical for (1) the evaluation of patients, (2) the monitoring of progression of the disease and (3) the adjustment of therapies.

Effort should be devoted to the selection of the sensors of interest for a given experiment in order to provide data in agreement with the clinical-functional assessment of tremor severity. Discrepancies are expected, but they should be as low as possible. Sensitive methods are required. Clinically-validated sensors small in size, light in weight and reconfigurable have the highest chances of becoming successful.

In addition to conventional sensors, new avenues of research are emerging to characterize and reduce tremor during daily activities. The so-called sensor fusion approach combines information from various sensors and might provide new ways to characterize oscillatory phenomena with direct clinical implications at middle term, including for BCIs. The field of wearable sensors is expanding quickly and there is a hope that body area network will greatly help the management of disabling tremor soon, using clusters of conventional or hybrid sensors.

## Figures and Tables

**Table 1.** Questionnaire of tremor-induced difficulties encountered during daily life (ADL-T24 score).

-to move a glass full of water on a table	
No problem	0
Slight difficulties	1
Important difficulties	2
Impossible	3

-to drink	
No problem	0
Slight difficulties	1
Important difficulties	2
Impossible	3

-to eat (use of forks and knives)	
No problem	0
Slight difficulties	1
Important difficulties	2
Impossible	3

-to shave	
No problem	0
Slight difficulties	1
Important difficulties	2
Impossible	3

-to write words on a sheet of paper or to sign	
No problem	0
Slight difficulties	1
Important difficulties	2
Impossible	3

-to read a book	
No problem	0
Slight difficulties	1
Important difficulties	2
Impossible	3

-to drive a car	
No problem	0
Slight difficulties	1
Important difficulties	2
Impossible	3

-to dress one-self	
No problem	0
Slight difficulties	1
Important difficulties	2
Impossible	3

Total Score:................................................../24	

**Table 2.** The composite clinical/neurophysiological/functional tremor evaluation scale (CNF-TES).

*C-TES (Clinical-TES)*
Anamnesis
Assessment of disability (Activities of Daily Living scales / ADL-T24)
Physical and Neurological examination
Tremor evaluation *- Body segments involved (head*, *trunk*, *upper limbs*, *lower limbs)* - Enhancing/reducing effect (effect of mental calculation/contralateral contractions) - Distribution (symmetry/asymmetry) - Grade/Amplitude - Frequency
Brain imaging (CT-scan - MRI - SPECT- PET)
Blood studies

*N-TES (Neurophysiological-TES)*
EMG and EEG recordings (time-frequency analysis, coherence, ERS/ERD)
Evaluation of pinch force
Analysis of writing (digitizing tablet)

*F-TES (Functional-TES; see* [13–14])
Mechanical counters
Box and block Test
9-Hole-Peg Test
Coin test

**Table 3.** Comparison of the most commonly used sensors for quantification and monitoring of tremor *.

	Assessment of kinematics**			EMG *Surface EMG (SEMG) Needle electrodes Fine-wire electrodes Long-term recordings*	Force transducers and force-feedback devices (haptic devices)
	Accelerometer	Gyroscope	Video		
Gravity effect influence	yes	no	no	no	no
Accuracy of frequency information	good	good	may be low	good	good
Signal-to-noise ratio	low to high	high	variable	high	high
Electrical contacts with subjects	no	no	no	yes	yes
Size	small	small	relatively large	small	Large
Painful	no	no	no	yes (needle EMG)	no
Cost	cheap	cheap	cheap to expensive	variable	expensive
Easy to use	yes	yes	yes	variable	relatively difficult
Data processing required	yes	yes	yes	yes	yes
Measurement of tremor amplitude	calculation from time/acceleration	measurement of inertial angular rate	from calibrated video frames	no	from force/mass or position encoder

* Emerging sensors include wearable textiles with integrated sensors, sensors based on the fusion approach and hybrid sensors.

** Includes also flexible angular sensors/goniometers and laser-based devices.

**Table 4.** Comparison of accelerometers.

**Parameter**	**Piezoelectric**	**Piezoresistive**	**Capacitive**
Gravitational component	No	Yes	Yes
Bandwidth	Wide	Low to moderate	Wide
Impedance	High	Low	Very high
Signal level	High	Low	Moderate
Ruggedness	Good	Moderate	Good
Cost	High	Low	High

Adapted from Wong *et al.*, 2007 [15].
